# Pharmacological effects of Niraparib and its combination with angiogenic inhibitor in ovarian cancer

**DOI:** 10.7150/jca.89082

**Published:** 2023-10-16

**Authors:** Huiwen Liu, Xue Chen, Chenpeng Tang, Xiangjian Luo

**Affiliations:** 1 Hunan Key Laboratory of Oncotarget Gene, Hunan Cancer Hospital and the Affiliated Cancer Hospital of Xiangya School of Medicine, Central South University, Changsha, Hunan 410013, PR China.; 2 Key Laboratory of Carcinogenesis and Invasion, Chinese Ministry of Education, Cancer Research Institute, School of Basic Medicine, Central South University, Changsha, Hunan 410078, PR China.; 3 Early Clinical Trial Center, Hunan Cancer Hospital and The Affiliated Cancer Hospital of Xiangya School of Medicine, Central South University, Changsha, Hunan 410013, PR China.

**Keywords:** Ovarian cancer, Niraparib, Brivanib, Regulated cell death

## Abstract

**Background:** Ovarian cancer (OC) represents the seventh most lethal female tumors worldwide. The combination of PARP inhibitor (PARPi) and angiogenic inhibitor has been shown to be effective as a first-line or second-line maintenance regimen to synergistically exert antitumor effects, which prompts us to further evaluate the therapeutic effect of the combination of PARP inhibitor Niraparib and anti-angiogenic Brivanib on OC.

**Method:**3-(4,5-dimethylthiazol-2-yl)-5-(3-carboxymethoxyphenyl)-2-(4-sulfophenyl)-2H-tetrazolium (MTS) assay were applied to evaluate the anti-proliferative effect of Niraparib, Brivanib, or the combination treatment on OC cells. The Annexin V-FITC/PI apoptotic assay was adopted to detect cell apoptosis. Tumor xenograft experiment and immunohistochemical (IHC) analysis were performed to evaluate the effect of single or combination treatment on the tumorigenicity of OC *in vivo*.

**Results:** Our current findings revealed that OC cells harboring BRAC1/2 mutations were more sensitive to Niraparib treatment compared to those with BRAC wild-type, and the addition of Brivanib enhanced programmed cell death (PCD) to sensitize OC cells with BRAC mutations to Niraparib treatment *in vitro and in vivo*.

**Conclusion:** Our work illustrates that the combination regimen of PARPi and angiogenic inhibitor treatment should be beneficial for the OC patients with BRAC mutations, at least partially owing to the induction of multiple forms of programmed cell death (PCD).

## Introduction

Ovarian cancer (OC) represents the seventh most lethal female tumors worldwide 1-5. The vast majority of OC patients exhibit recurrence and drug resistance within two years after surgery and platinum-containing chemotherapy 6.

BRCA1/2 is a tumor suppressor gene associated with homologous recombination and DNA damage repair. BRCA1 or BRCA2 mutations are mutually exclusive, and are usually called BRCA mutations indistinctively 7. The mutation carriers have a 25%-65% estimated lifetime risk of developing ovarian cancer 8. Poly ADP ribose polymerase (PARP) plays an important role in single strand break (SSB) repair and base excision repair (BER) pathways. After activation, PARP binds to DNA at the site of base excision and recruits other DNA repair proteins, including DNA ligase I, XRCC1 and DNA polymerase theta (POLQ) 9, 10. Recently, newly developed inhibitors targeting PARP-1 and PARP-2 can selectively kill ovarian cancer cells and prolong the overall survival of patients with this disease 11-15. Niraparib is a potent and highly selective PARP-1/2 inhibitor that has shown promising efficacy in patients with relapsed ovarian cancer. Niraparib was approved by the US Food and Drug Administration (FDA) in March 2017 for the maintenanced treatment of platinum-sensitive relapsed high-grade serous ovarian cancer. It displays 25-200 times more selective against cancer cells with BRCA1/2 mutation than those with wild-type 16.

Angiogenesis is closely related to tumor growth, proliferation and metastasis 17, 18. Brivanib is an orally administered selective inhibitor of the FGF and VEGF receptor families. Preclinical studies have shown that antiangiogenic drugs can affect homologous recombination repair (HRR) pathways in cancer cells. By inhibiting angiogenesis, antiangiogenic agents induce hypoxia in the tumor microenvironment and downregulate the key factors involved in HRR, such as BRCA1/2 and Rad51. In turn, PARP1 also plays a role in angiogenic process 19. The combination of PARPi and angiogenic inhibitor has been shown to be effective as a first-line or second-line maintenance regimen to synergistically exert antitumor effects in OC, breast cancer, (BC), colon cancer (CRC), non-small cell lung cancer (NSCLC), small cell lung cancer (SCLC) and prostate cancer (PC) 20, 21, which prompts us to further evaluate the therapeutic effect of the combination of PARP inhibitor Niraparib and anti-angiogenic Brivanib on OC.

Resistant to cell death is a hallmark of cancer. Regulated cell death (RCD) can be divided into multiple forms, mainly including apoptosis, necroptosis, autophagy, pyroptosis, and ferroptosis 22, 23. Necroptosis is a form of regulated cell death that critically depends on receptor interacting kinase 1 (RIPK1), RIPK3 and mixed lineage kinase domain like protein (MLKL) signaling. The interaction of RIPK1 and RIPK3 activates RIPK3, which further recruits and phosphorylates MLKL to induce necroptotic cell death 24, 25. Studies showed that RIPK3 can activate MLKL and the consequent necroptosis independent of RIPK1 24-26. In the present study, our findings manifest the synergistic antitumor effects of the newly FDA-approved PARP inhibitor Niraparib and the antiangiogenic Brivanib in BRCA1/2 mutant rather than BRCA wild-type OC cells, and that the activation of both apoptotic and necroptotic pathways were involved in the anti-tumor effect.

## Materials and methods

### Cell culture

Ovarian cancer PEO1 cells were maintained in RPMI-1640 media (Hyclone, UT, USA) containing 10% (v/v) heat-inactivated fetal bovine serum (FBS, Hyclone). Ovarian cancer UWB1.289 (CRL-2945) and UWB1.289+BRCA1 (CRL-2946) cells were grown in 50% RPMI-1640 Medium, 50% MEGM (Mammary Epithelial Growth Medium) supplemented with 5% (v/v) FBS (Hyclone). All the cell lines involved were cultured at 37°C in a humidified incubator containing 5% CO2.

### Reagents and antibodies

The antibodies against β-actin were purchased from Bioworld Technology. The antibodies against FGFR1, VEGFR2, cleaved-Caspase 3, cleaved-PARP were obtained from Cell Signaling Technologies (Danvers, MA, USA). The antibody to detect RIP3 was purchased from abcam (Cambridge, MA, USA). Anti-pMLKL was from ImmunoWay Biotechnology (Plano, TX, USA).

Niraparib was purchased from Selleck chemicals (Houston, TX, USA). Brivanib was from Zai Ding Pharmaceutical (Shanghai, China).

### Cell proliferation assay

Cells were evenly inoculated into a 96-well plate at a density of 6000 per well, and treated with corresponding concentrations of drugs for 24 h. Then CCK8 reagent was added and an enzyme-linked immunosorbent assay was used to detect cell proliferative activity.

### Western blotting

After electrophoretic separation and immunoblotting of whole-cell lysates, blots were incubated with corresponding primary antibodies and secondary antibodies labeled with horseradish peroxidase. Under the action of ECL Detection reagent, the destination stripes were visualized by using ChemiDoc XRS system and Image Lab software (Bio-Rad, California, USA).

### Annexin V-FITC/PI Apoptosis Assay

The Annexin V-FITC/PI apoptosis assay kit (4A biotech, Suzhou, China) was adopted to detect cell apoptosis. The procedure was performed as previously described 26, and FlowJo ver.10.0 software was applied for the analyses.

### Immunohistochemical (IHC) analysis

IHC analyses were essentially performed as previously described 27. The immune reactive score was calculated by multiplying the percentage of positive cells and staining intensity.

### Tumor xenograft studies

Female BALB/c nu/nu mice (5 weeks old) were subcutaneously inoculated with PEO1 cells (5×10^6^). After the tumors grew to a volume of about 80-100 mm^3^, mice were randomly divided into four groups (n =4 for each): (1) PBS, (2) gavage treatment with Brivanib (100mg/kg), (3) Niraparib (50mg/kg) (4) gavage treated with both Brivanib (100mg/kg) and Niraparib (50mg/kg) every day for 6 days, respectively. In each experiment, tumor volume was calculated according to the formula (V= length x width^2^/2). At the end of experiments, the mice were euthanized by CO2 inhalation and the tumors were stripped and weighed. Animal care experimental procedures were conducted in accordance with the approval of Xiangya hospital of Central South University (Changsha, China).

### Statistical analysis

All statistical calculations were conducted using SPSS ver.16.0. Differences between each group were analyzed by a two-tailed Student's t test.

## Results

### The addition of Brivanib sensitizes BRAC-mutated OC cells to Niraparib treatment

**Figure 1:**
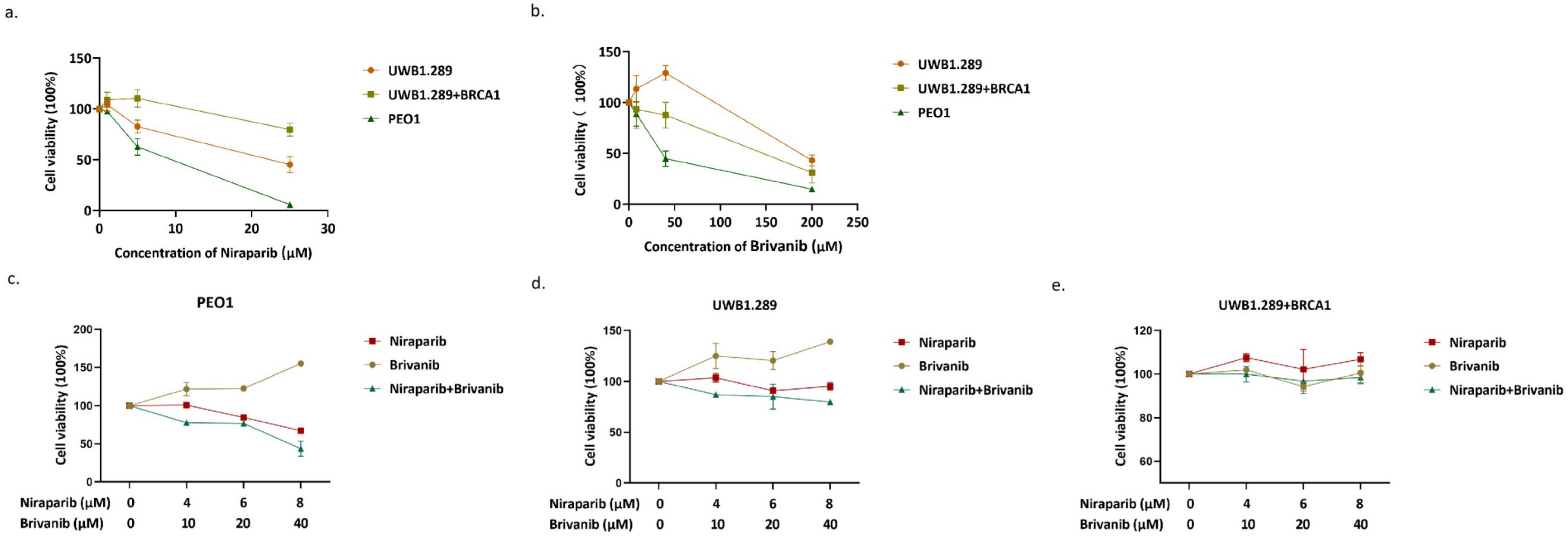
Addition of Brivanib sensitizes BRAC-mutated OC cells to Niraparib treatment. PEO1, UWB1.289 and UWB1.289+BRCA1 cells were treated with gradient concentrations of (a) Brivanib or (b)Niraparib for 24 h and analyzed by MTS assay. (c) PEO1, (d) UWB1.289, (e) UWB1.289+BRCA1 cells were treated with the combination of Niraparib and Brivanib for 24 h, and the cell viability was detected by MTS.

In order to determine whether the addition of Brivanib could enhance the anti-proliferative effect of Niraparib on OC cells harboring different *BRAC* gene status, OC cell lines UWB1.289 with BRAC1 mutation, PEO1 with BRAC2 mutation, and UWB1.289+BRCA1 with wild-type BRAC1 were adopted. First, we performed MTS assay at the concentration of 0,1,5,25 μM of Niraparib or 0, 8, 40, 200 μM of Brivanib in PEO1, UWB1.289, and UWB1.289+BRCA1 cells. The 50% inhibitory concentration (IC_50_) values of Niraparib were 7.487, 21.34, and 58.98 μM, and those of Brivanib were 41.54, 170.2, and 118.1 μM for PEO1, UWB1.289, and UWB1.289+BRCA1 cells, respectively (Figure 1a-1b). The results showed that PEO1 and UWB1.289 cells, which harbor BRAC1/2 mutations, were more sensitive to Niraparib treatment when compared to UWB1.289+BRCA1 with wild-type BRAC1. In addition, Brivanib treatment showed relatively lower IC_50_ value in PEO1. Next, to interrogate whether the combination of Niraparib with Brivanib existed synergistic effects, PEO1, UWB1.289, UWB1.289+BRCA1 cells were treated either with Niraparib alone or in combination with Brivanib at a series of concentrations for 24 h, and the cell viability was detected by MTS (Figure 1c-1e). The data manifested that the addition of Brivanib sensitized OC cells with BRAC mutations to Niraparib treatment, especially in PEO1 cells. However, no obviously synergistic effect has been observed in OC cells with wild-type BRAC.

### The addition of Brivanib enhances RCD of OC cells induced by Niraparib

**Figure 2:**
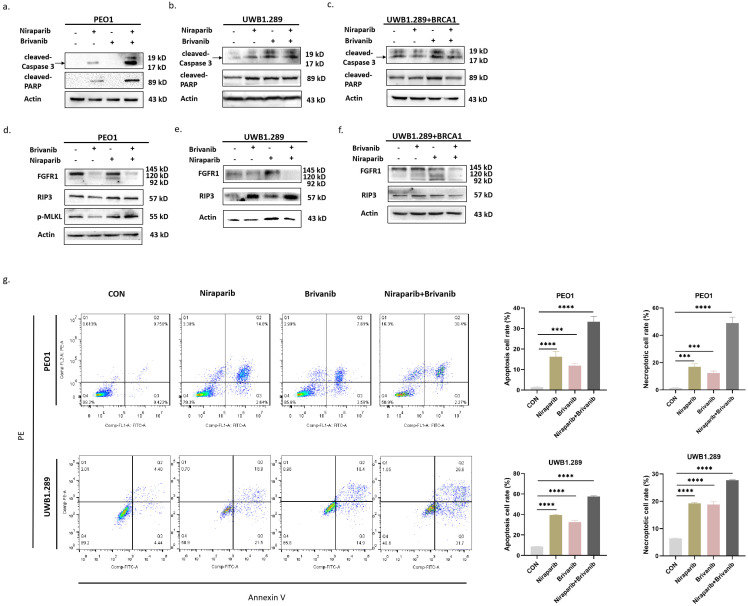
Immunoblotting analyses manifest that addition of Brivanib enhances RCD of OC cells induced by Niraparib. Cells were treated with Brivanib (40μM), or Niraparib (8μM), or the combination for 24 h. The protein levels of apoptotic markers cleaved-PARP and cleaved-Caspase 3 were detected by western blotting in (a) PEO1, (b) UWB1.289 and (c) UWB1.289+BRCA1 cells. The protein levels of angiogenic marker FGFR1 and necroptotic markers RIP3 and p-MLKL were detected by western blotting in (d) PEO1, (e) UWB1.289 and (f) UWB1.289+BRCA1 cells. Actin was used as a loading control. (g) PEO1 and UWB1.289 Cells were treated with Brivanib (40μM), or Niraparib (8μM), or the combination for 24 h, and then stained with annexin V/PE apoptotic detection kit and applied for flow cytometry analysis. The asterisks (***,****) indicate significant differences (p < 0.001).

In order to interrogate the underlying mechanism involved in the inhibitory effect of the combination of Niraparib and Brivanib treatment, we detected the expression of apoptotic markers cleaved-Caspase 3 and cleaved-PARP, necroptotic markers RIP3 and p-MLKL, as well as angiogenetic marker FGFR1 in PEO1, UWB1.289 and UWB1.289+BRCA1 cells. The data illustrated that compared to the groups treated by Niraparib or Brivanib alone, the expression levels of cleaved-PARP, cleaved-Caspase 3, RIP3 and p-MLKL were increased in BRAC-mutation OC cells, especially in PEO1 cells, but not so evident in BRAC-wild type cells (Figure 2a-2f). Additionally, the combination treatment markedly reduced FGFR1 expression in comparison with single Brivanib exposure (Figure 2d-2f). We further applied PEO1 and UWB1.289 cells for flow cytometry analyses after treatment with Niraparib or Brivanib or the combination for 24 h. The results showed that compared with Niraparib or Brivanib treatment alone, the combination significantly up-regulated both apoptotic and necroptotic rates of OC cells with BRAC mutations (Figure 2g), which was in accordance with those of immunoblotting analyses. Altogether, these data indicate that the addition of anti-angiogenic Brivanib enhances the apoptotic and necroptotic cell death induced by Niraparib treatment in OC cells harboring BRAC mutations.

### The addition of Brivanib enhances the inhibitory effect of Niraparib on OC *in vivo*

**Figure 3:**
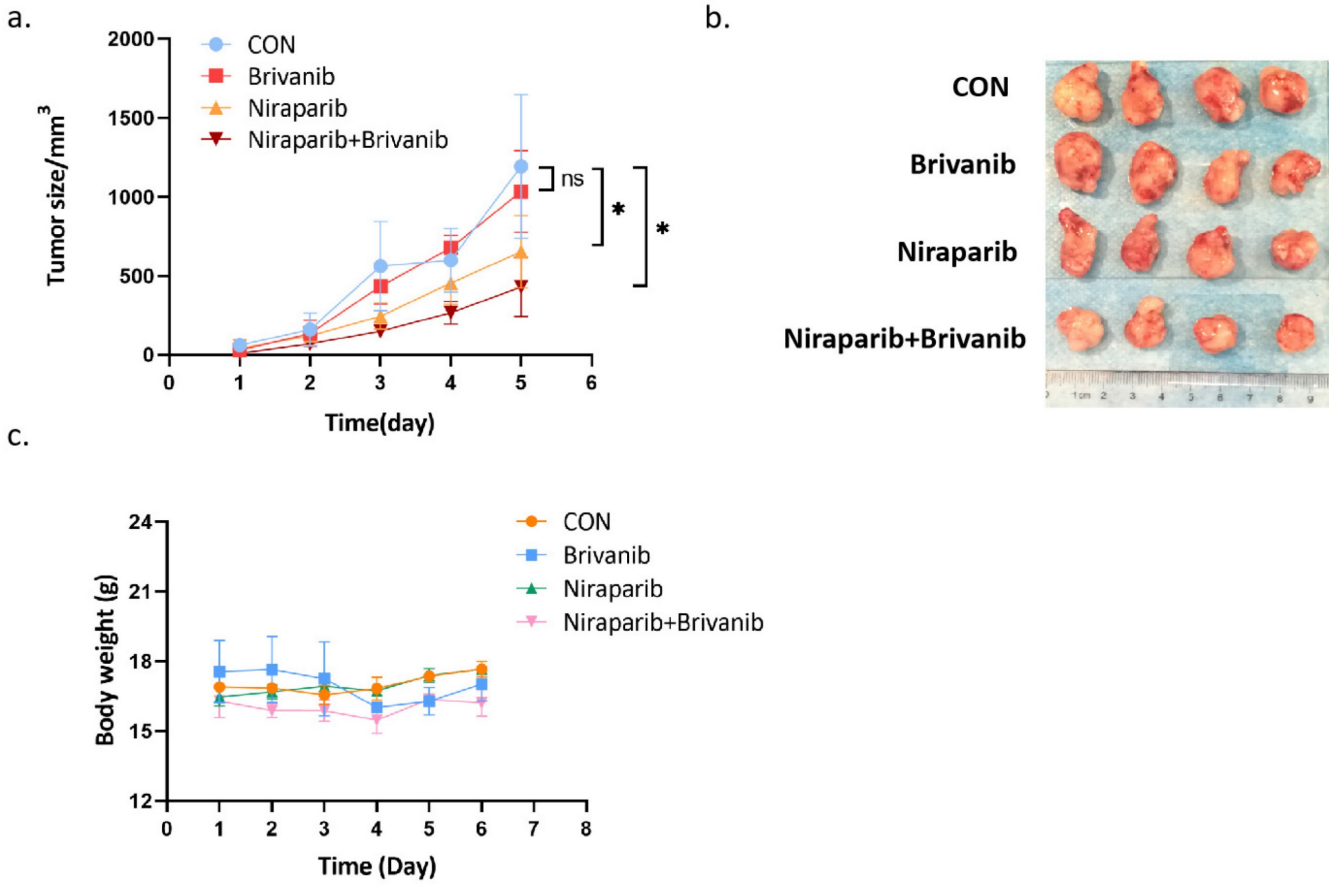
Addition of Brivanib enhances the inhibitory effect of Niraparib on OC *in vivo*. Athymic BALB/c nude mice bearing PEO1 cells were randomly separated into 4 groups (n = 4) and gavage administrated with PBS (CON), Brivanib (100mg/kg), Niraparib (50mg/kg) or Niraparib (50mg/kg) combined with Brivanib (100mg/kg) every day for 6 days. (a) Tumor volume was observed and calculated according to the formula (V = length x width^2^/2). (b) At the end of the experiment, the mice were sacrificed and the tumors were separated. (c) During the experiment, body weight of the mice in each group was monitored and shown in the graph. The asterisks (*) indicate significant differences (p < 0.05).

**Figure 4:**
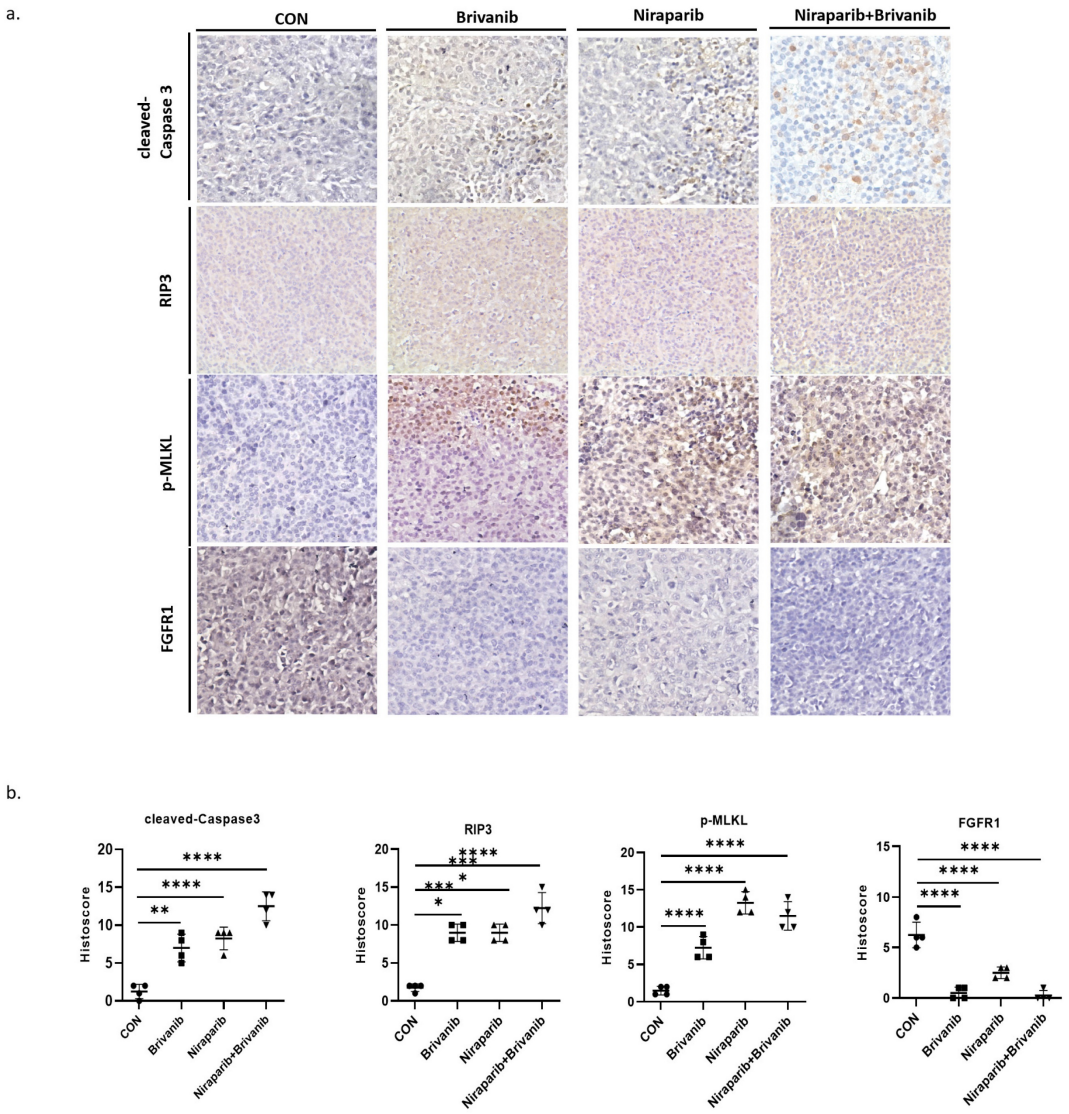
Immunohistochemistry analyses manifest the occurrence of RCD in OC tissues with the combination treatment of Niraparib and Brivanib. (a) Representative images of tumor sections in the designated groups stained with indicated antibodies. Antibody staining is in brown and nuclear counter staining is in blue. (b) Scatter diagram showed Histoscore for the indicated antibody staining in tumor samples. The asterisks (*,**,***) indicate significant differences (p < 0.05, p < 0.01, p < 0.001, respectively).

To further assess the effect of the combination treatment on OC *in vivo*, we performed a xenograft experiment using PEO1, which is a representative of OC cells with BRAC mutation. PEO1 cells were injected subcutaneously into BABL/c nu/nu mice to establish a xenograft tumor model. Athymic BALB/c nude mice bearing PEO1 cells were randomly separated into 4 groups and were gavage administrated with phosphate-buffered saline (PBS), Niraparib, Brivanib or Niraparib combined with Brivanib every day. Combined administration of Niraparib and Brivanib resulted in a significant inhibition of tumor load compared to the vehicle group (Figure 3a-3b), while no obvious side effects have been observed (Figure 3c). Moreover, immunohistochemistry (IHC) staining of cleaved-Caspase-3, RIP3, p-MLKL, and FGFR1 demonstrated that the combination administration remarkably induced apoptosis in OC cells relative to Niraparib or Brivanib treatment alone, or the vehicle control. The increased expression of RIP3 and p-MLKL in the combination group manifested that the necroptotic pathway was activated (Figure 4). In addition, Niraparib or Brivanib treatment alone, or the combination inhibited FGFR1 expression effectively (Figure 4), which suggested that PARP inhibitor (PARPi) might also exhibit anti-angiogenic activity *in vivo*. Besides, we also performed xenograft experiments using UWB1.289 and UWB1.289+BRCA1 cells. Although Niraparib and Brivanib combination treatment resulted in reasonable growth inhibition in UWB1.289 xenografts compared to the control or single treatment group, in general, the tumorigenicity of UWB1.289 cells were inferior (Supplementary figure 1); and UWB1.289+BRCA1 cells showed non-tumorigenic.

Therefore, these results indicate that Brivanib enhances the inhibitory effect of Niraparib on OC harboring BRAC mutation *in vivo*, and the activation of multiple PCD pathways are involved in this process.

## Discussion

The combination of PARPi and anti-angiogenic treatment has been shown to be a synergistic antitumor regimen for the first-line or second-line maintenance treatment of OC. Among PARPis, Olaparib, Niraparib and Rucaparib have been approved for the treatment of recurrent OC, Talazoparib and Veliparib are currently in early-stage trials 28. Olaparib in combination with the anti-angiogenic drug bevacizumab has been approved for the first-line standard therapy for OC patients with BRCA-mutated or homologous recombination deficiency (HRD), and can significantly improve the progression-free survival of patients 29, 30. Besides, the combination of tumor treating fields (TTF) and a PARP inhibitor during radiotherapy had a synergistic effect on tumor restriction and was well tolerated by patients 31. However, the effect of the combination of Niraparib with angiogenic inhibitor Brivanib on tumor progression has not been reported yet. Brivanib is the first-in-class FGF/VEGF inhibitor currently in late-stage clinical trials, blocking VEGF receptors 1-3 and FGF receptors 1-2 against angiogenic activity and inhibiting FGF-dependent tumor growth 32. It has been reported to exert antitumor activity in clinical trial subjects with hepatocellular carcinoma, colorectal cancer, and soft tissue sarcoma, and is currently under development for the Chinese pharmaceutical market 33. In the present study, we illustrate that the combination of Niraparib with Brivanib effectively inhibites the proliferation of OC *in vitro* and *in vivo*.

## Conclusion

Our work supports that the combination regimen of PARPi and angiogenic inhibitor treatment should be beneficial for the OC patients with BRAC mutations, at least partially owing to the induction of multiple forms of PCD.

BRCA1 and BRCA2 mutations are the most significant molecular abnormality in OC by far and have been shown to be a valuable prognostic and predictive indicator after chemotherapy 34. OC harboring BRCA mutation have been reported to be featured with some unique characteristics, including a higher sensitivity to PARP inhibitors 35, which is consistent with our results. Moreover, we found that the addition of Brivanib augmented the induction of apoptosis and necroptosis of OC cells caused by Niraparib treatment. *In vivo* tumorigenic experiments further supported that the combination of Niraparib and Brivanib administration effectively reduced tumor volume and activated multiple programmed cell death (PCD) pathways, effectively hampering the tumor growth of OC with BRAC mutation. Whereas, it should be pointed out that there are still shortcomings in our study, and the therapeutic effect of combination Niraparib with angiogenic inhibitor in clinical OC patients needs further investigation. To define the candidate OC patient groups that would benefit for PARP inhibitor therapies alone or in combination with antiangiogenic treatment, taking into consideration the genetic background, tumor biomarkers, and so on, should be one of the future research directions.

## Supplementary Material

Supplementary materials and methods, figure.
