# Supplementary material for: Pharmacological effects of Niraparib and its combination with angiogenic inhibitor in ovarian cancer
Source: J Cancer. 2023 Oct 16;14(18):3397–403. doi: 10.7150/jca.89082 (PMC10647199; doi:10.7150/jca.89082)
Supplement: Supplementary file 1 — Supplementary materials and methods, figure. [file jcav14p3397s1.pdf]

Supplemental Materials and Methods

Tumor xenograft studies

Female BALB/c nu/nu mice (5 weeks old) were subcutaneously inoculated with UWB1.289 (1×10^7) or UWB1.289+BRCA1 cells (1×10^7), respectively. After the tumors grew to a volume of about 80–100 mm^3, mice were randomly divided into four groups (n =4 for each): (1) PBS, (2) gavage treatment with Brivanib (100mg/kg), (3) Niraparib (50mg/kg) (4) gavage treated with both Brivanib (100mg/kg) and Niraparib (50mg/kg) every other day for 3 weeks, respectively. In each experiment, tumor volume was calculated according to the formula ($V= length \times width^2/2$). At the end of experiments, the mice were euthanized by CO2 inhalation and the tumors were stripped and weighed. Animal care experimental procedures were conducted in accordance with the approval of Xiangya hospital of Central South University (Changsha, China).

Supplementary figure legends

Supplementary figure 1 Athymic BALB/c nude mice bearing UWB1.289 cells were randomly separated into 4 groups (n = 4) and gavage administrated with PBS (CON), Brivanib (100mg/kg), Niraparib (50mg/kg) or Brivanib (100mg/kg) combined with Niraparib (50mg/kg) every other day for 3 weeks, respectively. At the end of the experiment, the mice were sacrificed and the tumors were separated.

UWB1.289

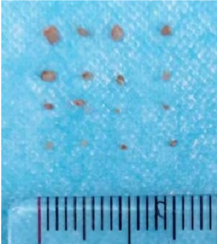

CON

Brivanib

Niraparib

Niraparib+Brivanib
